# Neutrophil–Lymphocyte and Platelet–Lymphocyte Ratios in Distinguishing Lung Cancer in People with HIV

**DOI:** 10.1155/2024/8822024

**Published:** 2024-01-23

**Authors:** Joseph Baruch Baluku, Sharon Namiiro, Brenda Namanda, Martin Nabwana, Irene Andia-Biraro, William Worodria, Robert Salata, Sayoki Mfinanga, Stanton Gerson, Bruce Kirenga

**Affiliations:** ^1^Makerere University Lung Institute, Kampala, Uganda; ^2^Division of Pulmonology, Kiruddu National Referral Hospital, Kampala, Uganda; ^3^Department of Internal Medicine, Makerere University College of Health Sciences, Kampala, Uganda; ^4^Makerere University–Johns Hopkins University Research Collaboration, Kampala, Uganda; ^5^Department of Medicine, UH Cleveland Medical Center, Cleveland, USA; ^6^Muhimbili Center, National Institute for Medical Research, Dar es Salaam, Tanzania; ^7^School of Medicine, Case Western Reserve University, Cleveland, USA

## Abstract

**Objective:**

The neutrophil–lymphocyte ratio (NLR) and platelet–lymphocyte ratio (PLR) demonstrate good diagnostic accuracy in distinguishing lung cancer patients from healthy individuals, primarily in HIV-negative populations. We determined the sensitivity (Se), specificity (Sp), and area under the curve (AUC) of the NLR and PLR in discriminating between people living with HIV (PLWH) with and without lung cancer.

**Methods:**

This is a comparative analysis of secondary data. Cases were PLWH with lung cancer from a retrospective cohort treated at the Uganda Cancer Institute. Controls were unmatched PLWH without lung cancer who were randomly selected from three HIV clinics in Uganda. Se, Sp, and AUC analysis and determination of optimal cutoffs were performed using receiver operating characteristic (ROC) curves.

**Results:**

Of 115 PLWH (18 cases and 97 controls), 83 (72.2%) were female, 110 (95.7) were on ART, and the median (IQR) age was 46 (38–51) years. The median (IQR) NLR was higher among cases than controls (3.53 (3.14–7.71) vs. 0.92 (0.67–1.09), *p*  < 0.001). Similarly, the PLR was higher among cases than controls (237.5 (177.8–361.6) vs. 123.6 (100.6–155.4), *p*=0.001). At a cutoff of 2.44, the respective Se, Sp, and AUC of the NLR were 87.5% (95% CI: 61.7%–98.4%), 100% (95% CI: 96.2%–100%), and 0.94 (95% CI: 0.85–1.00, *p*  < 0.001). Similarly, the respective Se, Sp, and AUC for the PLR were 75% (95% CI: 47.6%–92.7%), 87.2% (95% CI: 78.8%–93.2%), and 0.81 (95% CI: 0.70–0.93, *p*  < 0.001) at a cutoff of 196.3.

**Conclusion:**

The NLR and PLR discriminated PLWH with and without lung cancer and could be useful in PLWH with respiratory symptoms in whom lung cancer can easily be misdiagnosed as other lung pathology.

## 1. Introduction

In 2020, ∼2.2 million new lung cancer cases were reported, along with 1.8 million related deaths [1]. Between 1990 and 2019, the number of lung cancer deaths increased by 92%, primarily due to aging and population growth [2]. HIV infection increases one's risk for lung cancer by about 1.3–4 times the average risk in the general population [3, 4]. In fact, lung cancer is the leading cause of cancer-related deaths among people living with HIV (PLWH) in several cohorts [5–7]. The high risk of lung cancer among PLWH is attributed to various factors including high rates of smoking, oncogenic HIV proteins, chronic inflammation resulting from recurrent opportunistic infections, and increasing longevity due to effective antiretroviral therapy [8]. PLWH with lung cancer have a fourfold higher risk of dying compared to HIV-negative individuals with the same diagnosis [9]. Screening for lung cancer with low-dose computed tomography (LDCT) among high-risk PLWH has the potential to detect lung cancer early and reduce mortality by almost 20% [10, 11]. However, there are no straightforward screening strategies among PLWH. While LDCT screening has the potential to detect lung cancer early and reduce mortality, it is costly and largely unavailable in low-income settings where the majority of PLWH reside. This highlights the need for affordable and accessible point-of-care biomarkers for early lung cancer diagnosis in this population.

The neutrophil–lymphocyte ratio (NLR) and the platelet–lymphocyte ratio (PLR) have demonstrated good diagnostic accuracy in differentiating lung cancer patients from healthy controls, although primarily in studies involving HIV-negative individuals [12–16]. These ratios are established markers of systemic inflammation, typically elevated in malignancies and other disease conditions [17–22]. However, their potential for diagnosing lung cancer in PLWH remains underexplored.

The objective of this analysis was to determine the sensitivity (Se), specificity (Sp), and area under the curve (AUC) of the NLR and PLR in discriminating between PLWH with and without lung cancer.

## 2. Methods

### 2.1. Study Design, Population, and Setting

This comparative analysis involves secondary data from a cohort of PLWH with lung cancer (cases) [23] and a cross-sectional study of randomly selected PLWH without lung cancer (controls) [24]. Cases were PLWH with histologically confirmed primary lung cancer who received treatment from the Uganda Cancer Institute (UCI) between 2008 and 2018. Controls were apparently healthy PLWH who were randomly selected from three HIV clinics in Uganda. The study methods of the primary studies are described elsewhere [23, 24]. Briefly, a census of cases with HIV-related cancer was conducted from medical records at the UCI. The eligibility criteria were PLWH with histological confirmation of primary lung cancer at UCI between 2008 and 2018. Demographic data, medical history and laboratory results were abstracted using a data abstraction form. Controls were randomly selected from the OpenEMR system, a medical records software used at the HIV clinics. Eligible participants were adult PLWH receiving ART at Kiruddu National Referral Hospital, St. Francis Nsambya Hospital, and Mbarara Regional Referral Hospital in Uganda. The variables of interest are described below.

### 2.2. Data Collection and Study Measurements

For the cases, data were abstracted from treatment records using a data abstraction form. Pretreatment sociodemographic (age, sex, smoking history, and alcohol use) and clinical data (cancer stage, performance status, and antiretroviral therapy (ART) treatment details) were obtained. Pretreatment full blood counts and other laboratory test findings (lactate dehydrogenase, liver transaminases, serum creatinine, urea, albumin, and bilirubin) were abstracted as well. For the controls, data were obtained using a study questionnaire through a face-to-face interview, and additional data on ART treatment status were confirmed from the treatment records. Blood samples were tested for the full hemogram and the other laboratory tests above.

The NLR and PLR were calculated by dividing the absolute neutrophil and platelet counts by the lymphocyte counts from the full hemogram report, respectively. The study outcomes were the Se, Sp, and AUC of the NLR and PLR.

### 2.3. Statistical Analysis

Data were analyzed with Stata 17.0. We compared categorical variables between cases and controls using Pearson's *χ*^2^ test and continuous variables using Mood's median test. We further constructed an ad hoc multivariable robust Poisson regression model that controlled for the NLR and PLR to determine factors that are independently associated with having lung cancer. We determined the optimal cutoffs, Se, Sp and AUC of the NLR and PLR using receiver operating characteristic (ROC) curves. In this analysis, having lung cancer was considered to be the positive comparator. The optimal cutoff was the point on the ROC curve that gave the maximal Youden index [25]. The NLR and PLR were considered to have discriminating ability between cases and controls, if the AUC was significantly different from the null value of 0.5 (null: AUC = 0.5). Statistical significance was set at *p*  < 0.05.

## 3. Results

### 3.1. Study Participant Characteristics

The study included 18 cases (PLWH with lung cancer) and 97 controls (PLWH without lung cancer). Characteristics of cases and controls are shown in Table 1. Nonsmall cell lung cancer was the predominant histological type observed in 17 (94.4%) cases and the majority had stage IV disease (88.9%). Cases had a lower body mass index, lymphocyte counts, hemoglobin level, serum creatinine, and lactate dehydrogenase levels. Further, a lower proportion of cases than controls were on ART (72.2% vs. 100.0%, *p*  < 0.001), and the median (IQR) CD4 count was lower among cases than controls (380 (244–595) vs. 956 (745–1,251) cells/mm^3^, *p*  < 0.001). Additionally, cases exhibited higher counts of leucocytes, neutrophils, and basophils. There was a higher proportion of cases than controls who reported respiratory symptoms and a previous tuberculosis episode (cured or completed treatment). Cough was the most common symptom (12.4% in controls vs. 72.2% in cases, *p*  < 0.001). Among the controls, 88 (91.7%) were virally suppressed and 72 (74.2%) had World Health Organization HIV stage I and II disease, but these data were not available for cases. However, at multivariable analysis (Table 2), there were no significant differences between cases and controls.

### 3.2. Sensitivity, Specificity, and Area under the Curve of the NLR and PLR

Cases exhibited significantly higher NLR and PLR values compared to controls. That is, cases had a higher median (IQR) NLR than controls (3.53 (3.14–7.71) vs. 0.92 (0.67–1.09), *p*  < 0.001). Similarly, the PLR was higher among cases than controls (237.5 (177.8–361.6) vs. 123.6 (100.6–155.4), *p*=0.001).

**Figure 1:**
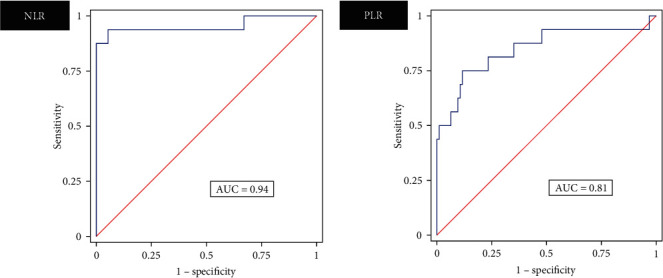
Receiver operating characteristic (ROC) curves for sensitivity vs. 1−specificity of the neutrophil–leucocyte ratio (NLR) and platelet–leucocyte ratio (PLR). Area under the curve (AUC).

At a cutoff of 2.44, the respective Se, Sp, and AUC of the NLR were 87.5% (95% confidence interval (CI): 61.7%–98.4%), 100% (95% CI: 96.2%–100%), and 0.94 (95% CI: 0.85–1.00, *p*  < 0.001). Similarly, the respective Se, Sp, and AUC for the PLR were 75% (95% CI: 47.6%–92.7%), 87.2% (95% CI: 78.8%–93.2%), and 0.81 (95% CI: 0.70–0.93, *p*  < 0.001) at a cutoff of 196.3.  Figure 1 shows the ROC curves.

## 4. Discussion

This study assessed the performance of NLR and PLR in differentiating PLWH with lung cancer from those without. The NLR had a high accuracy with the respective Se, Sp, and AUC of 88%, 100%, and 0.94 at a cutoff of 2.44. Similarly, the PLR had a relatively high accuracy with a respective Se, Sp, and AUC of 75%, 87%, and 0.81 at a cutoff of 196.3. The findings suggest that NLR and PLR are promising biomarkers for distinguishing PLWH with and without lung cancer. Nevertheless, the advanced stage of cancer in the study cases (all had stage 3 and above) limits generalizability to subtle disease detection in a broader PLWH population. Nonetheless, they can be useful in PLWH with respiratory morbidity in whom lung cancer can easily be misdiagnosed as pulmonary tuberculosis or other lung pathology. In our study, a higher proportion of cases than controls reported previous TB treatment, although it is uncertain from these data whether this was bacteriologically confirmed TB or misdiagnosis of lung cancer. This association underscores the need for thorough respiratory evaluations in PLWH, especially those with a history of tuberculosis, as they might be at an increased risk of developing lung cancer or be misdiagnosed as having pulmonary TB instead of lung cancer.

Other studies have also reported a high accuracy of the NLR and PLR in advanced lung cancer among HIV negative individuals, which further emphasizes the potential use in people with existent but confusing symptomatology [12]. As expected, the cases in our study had more respiratory symptoms than controls. The results from our study need to be validated in a larger study to increase the certainty of the usefulness of these biomarkers. It would be important to see if the ratios perform well in PLWH with subclinical lung cancer. Unfortunately, as observed in our study, PLWH with lung cancer often present with advanced lung cancer even in high-income settings [26].

Similar to our study, several studies have demonstrated the diagnostic utility of the NLR and PLR in discriminating HIV negative people with and without lung cancer, albeit at different cutoffs. Nikolić et al. [13] reported a respective Se and Sp of the NLR (cutoff of 2.71) and PLR (cutoff of 182.31) of 77% and 87%, and 51% and 91%. Furthermore, Gupta et al. [14] reported the respective Se and Sp of the NLR (cutoff of 2.5) and PLR (cutoff of 148.7) of 75% and 86%, and 48% and 88%. However, Zhu et al. [16] reported a modest accuracy with respective AUC of the NLR and PLR of 0.684 and 0.623. While it is evident from these studies and ours that the NLR has a better diagnostic accuracy than the PLR, straightforward comparisons cannot be made because our population is PLWH. It might be the case that these ratios have better diagnostic performance in PLWH because of the already ongoing systemic inflammation that may be accentuated by neoplasm. Indeed abnormalities in the complete blood count parameters are commoner in PLWH due to the direct effect of HIV infection, whereby it infects the multipotent hemopoietic stem cells and alters the bone marrow microenvironment [27]. Other mechanisms include the effect of opportunistic infections, nutritional deficiencies, immune-mediated destruction of blood cells and cancer [27].

Besides the NLR and PLR findings, the study identified several characteristics associated with lung cancer in PLWH, including lower body mass index, lymphocyte counts, hemoglobin levels, serum creatinine, and lactate dehydrogenase. These findings suggest that PLWH with such characteristics are more likely to have lung cancer and may benefit from closer monitoring or targeted screening strategies, although this needs to be validated by larger studies. Additionally, the higher counts of leucocytes, neutrophils, and basophils in lung cancer cases could be a response to cancer or an indicator of underlying inflammation. The lower median CD4 count in lung cancer patients is another observation in our study which could be attributed to the lower ART coverage among the cases compared to the controls. While it is wellknown that HIV lowers CD4 T-cell counts, which potentially affects immune surveillance and cytotoxicity against cancer cells, the association between low CD4 counts and lung cancer is not consistently observed [28, 29]. Notwithstanding, ensuring ART coverage for all PLWH is crucial for overall survival of PLWH and lung cancer [30–32].

A major drawback to our study is the small sample size that explains the wide confidence intervals. The small sample size necessitates further research with larger cohorts to validate these preliminary findings. The analysis is also limited by the lack of data on viral suppression and WHO HIV stage for lung cancer cases.

## 5. Conclusion

Findings from this preliminary study indicate that NLR is highly accurate; whereas, PLR shows moderate accuracy in distinguishing PLWH with lung cancer from those without. The utility of these ratios is likely to be among PLWH with overt but confusing respiratory symptoms. A larger study is needed to validate our findings.

## Figures and Tables

**Table 1:** Characteristics of PLWH with and without lung cancer.

Characteristic	Total *N* = 115 (%)	Controls *n* = 97 (%)	Cases *n* = 18 (%)	*p* Value
Age, median (IQR), years	46 (38, 51)	46 (38, 51)	49.5 (40, 56)	0.219
Body mass index, median (IQR), kg/m^2^	26.7 (22.7, 31.2)	26.7 (23.0, 31.6)	20.8 (19.0, 23.2)	**0.006**
Females	83 (72.2)	72 (74.2)	11 (61.1)	0.254
Patient on antiretroviral therapy	110 (95.7)	97 (100)	13 (72.2)	**<0.001**
Previous tuberculosis treatment	26 (22.6)	16 (16.5)	10 (55.6)	**<0.001**
Any history of smoking	18 (15.7)	13 (13.4)	5 (27.8)	0.155
History of alcohol use	63 (54.8)	53 (54.6)	10 (55.6)	0.943
Current symptoms				
Cough	25 (21.9)	12 (12.4)	13 (76.5)	**<0.001**
Weight loss	16 (13.9)	3 (3.1)	13 (72.2)	**<0.001**
Chest pain, *n = 111*	18 (16.2)	7 (7.2)	11 (78.6)	**<0.001**
Hemoptysis	5 (4.4)	0 (0)	5 (29.4)	**<0.001**
Laboratory results, median (IQR)				
Leucocyte count (×10^9^ per liter)	5.1 (4.0, 5.8)	4.8 (3.9, 5.7)	5.9 (5.5, 8.6)	**0.016**
Neutrophil count (×10^9^ per liter)	2.1 (1.6, 3.1)	2.0 (1.5, 2.5)	4.4 (3.2, 6.8)	**0.003**
Lymphocyte count (×10^9^ per liter)	2.1 (1.6, 2.5)	2.3 (1.8, 2.6)	1.1 (0.8, 1.8)	**<0.001**
Eosinophil count (×10^9^ per liter)	0.12 (0.06, 0.21)	0.12 (0.07, 0.21)	0.10 (0.02, 0.27)	0.813
Basophil count (×10^9^ per liter)	0.01 (0.01, 0.02)	0.01 (0.01, 0.02)	0.03 (0.00, 0.06)	**0.001**
Neutrophil–leucocyte ratio	0.98 (0.72, 1.68)	0.92 (0.67, 1.09)	3.52 (3.14, 7.71)	**<0.001**
Platelet–leucocyte ratio	128.4 (101.5, 167.5)	123.6 (100.6, 155.4)	237.5 (177.8, 361.6)	**0.001**
Hemoglobin level	14.2 (13.2, 15.2)	14.4 (13.5, 15.5)	11 (9.5, 12.5)	**<0.001**
Mean corpuscular volume (g/dL)	91.3 (86.1, 95.9)	913 (87.3, 95.8)	91.2 (80.5, 101.0)	0.937
Platelet count (cells/mm^3^)	264.9 (221.9, 309.7)	263.5 (221.9, 301.8)	286 (222, 371)	0.761
Creatinine (mmol/L)	104.0 (86.4, 121.6)	107.1 (92.6, 123.7)	66 (57, 83.5)	**0.001**
Total bilirubin (*µ*mol/L)	0.39 (0.24, 0.65)	0.35 (0.23, 0.55)	4.6 (2.7, 9.7)	**<0.001**
Alkaline aminophosphatase (IU/L)	110 (82, 150)	110 (81, 147)	137 (95, 202.6)	0.365
Alanine aminotransferase (IU/L)	20.3 (15.4, 28.1)	20.6 (15.5, 28.1)	17.8 (13.6, 27.3)	0.616
Aspartate aminotransferase (IU/L)	27.1 (22.5, 33.5)	27.3 (22.7, 33.5)	25.1 (16.7, 37.8)	0.616
Gamma-glutamyl transferase (IU/L)	47.1 (35, 66.1)	46.7 (33.6, 61.2)	76.8 (44, 121)	0.140
Lactate dehydrogenase (IU/L)	564 (411.6, 772)	594 (450, 780)	248.8 (185, 356)	**0.002**

Bold *p*-values indicate a statistically significant result.

**Table 2:** Factors associated with lung cancer among PLWH.

Characteristic	Crude RR (95% CI)	*p* Value	Adjusted RR (95% CI)	*p* Value
Patient on ART				
No	Ref		Ref	
Yes	0.12 (0.04, 0.33)	**<0.001**	1.53 (0.30, 7.25)	0.608
Previous TB treatment				
No	Ref		Ref	
Yes	4.28 (1.69, 10.84)	**0.002**	1.83 (0.46, 7.25)	0.388
Cough				
No	Ref		Ref	
Yes	11.57 (3.77, 35.48)	**<0.001**	2.29 (0.37, 14.12)	0.374
Weight loss				
No	Ref		Ref	
Yes	16.09 (5.74, 45.12)	**<0.001**	1.27 (0.10, 16.02)	0.852
Chest pain				
No	Ref			
Yes	18.94 (5.29, 67.91)	**<0.001**		
Laboratory results				
White blood cell count	1.13 (1.06, 1.19)	**<0.001**	1.95 (0.35, 10.76)	0.442
Neutrophil count	1.15 (1.08, 1.22)	**<0.001**	0.50 (0.10, 2.57)	0.407
Lymphocyte count	0.23 (0.11, 0.50)	**<0.001**	0.27 (0.02, 3.53)	0.320
Hemoglobin level	0.57 (0.46, 0.70)	**<0.001**	0.74 (0.50, 1.08)	0.121
Creatinine	0.97 (0.96, 0.99)	**0.001**	0.99 (0.97, 1.01)	0.177
NLR	1.13 (1.07, 1.19)	**<0.001**	0.99 (0.73, 1.36)	0.959
PLR	1.003 (1.001, 1.004)	**<0.001**	1.00 (0.10, 1.01)	0.377

ART, antiretroviral therapy; NLR, neutrophil–leucocyte ratio; PLR, platelet–leucocyte ratio. Bold *p*-values indicate a statistically significant result.

## Data Availability

Datasets used in this analysis are available from the corresponding author upon reasonable request.
